# Association between elevated central venous pressure and outcomes in critically ill patients

**DOI:** 10.1186/s13613-017-0306-1

**Published:** 2017-08-09

**Authors:** Dong-kai Li, Xiao-ting Wang, Da-wei Liu

**Affiliations:** Department of Critical Care Medicine, Peking Union Medical College Hospital, Peking Union Medical College, Chinese Academy of Medical Science, 1 Shuaifuyuan, Dongcheng District, Beijing, 100730 China

**Keywords:** Central venous pressure, Survival, Critical care

## Abstract

**Background:**

Some prior studies have shown that elevated mean central venous pressure in certain patient populations and disease processes may lead to poor prognosis. However, these studies failed to generalize the concept of elevated central venous pressure (ECVP) load to all patients in critical care settings because of the limited cases and exclusive cohorts. The aim of the study was to investigate the association between elevated central venous pressure and outcomes in critical care.

**Methods:**

We performed a retrospective analysis on a single-center public database (MIMIC) of more than 9000 patients and more than 500,000 records of central venous pressure measurement. We evaluated the association between mean central venous pressure level and 28-day mortality after intensive care unit admission. The secondary outcomes were duration of mechanical ventilation, vasoactive drug use, laboratory results related to organ dysfunction and length of intensive care unit hospitalization. Accordingly, we proposed the concept of ECVP_10_ (the time sum of CVP above 10 mmHg) and investigated its association with outcome.

**Results:**

There were 1645 deaths at 28 days after admission. Compared with the lowest quartile of mean central venous pressure [mean (SD) 7.4 (1.9) mmHg], the highest quartile [17.4 (4.1) mmHg] was associated with a 33.6% (95% CI 1.117–1.599) higher adjusted risk of death. Poor secondary outcomes were also associated with higher quartiles of elevated mean central venous pressure. After stratification by mean central venous pressure, elevated duration of central venous pressure above 10 mmHg was significantly higher in the non-survival group than in the survival group.

**Conclusions:**

Elevated central venous pressure level correlated with poor outcomes and prolonged treatment in critical care settings. Level and duration of elevated central venous pressure should be both evaluated to establish its cause and apply appropriate treatment.

**Electronic supplementary material:**

The online version of this article (doi:10.1186/s13613-017-0306-1) contains supplementary material, which is available to authorized users.

## Background

Elevated right atrial pressure or central venous pressure (CVP) occurs frequently in critical care settings [1–4] and may be caused by several conditions, such as congestive heart failure syndrome, constrictive pericardial disease, tension pneumothorax, and resuscitation/evacuation phases of septic shock. Although CVP has been utilized as a surrogate of intravascular volume in critically ill patients, recent studies have challenged the validity of elevated CVP (ECVP) in critical care settings [5–9]. Based on rationale provided by the Starling curves and Guyton model on cardiac function, CVP is determined by the interaction of cardiac function and the return of blood to the heart. An ECVP might indicate an impediment to the venous return and microcirculatory blood flow as well as accompanying lung edema and splanchnic congestion [3, 10], which may further worsen the potential organ failure in critical patients.

Some prior studies have shown that increased mean CVP in certain patient populations and disease processes may lead to poor prognosis. In septic patients, increased mean CVP was associated with worse outcome, including increased 28-day mortality as well as the development and progression of acute kidney injury [6, 7, 11]. For patients with cardiovascular diseases, ECVP might be associated with impaired hepatic and renal function and may be independently related to all-cause mortality [8, 9]. These studies, however, could not generalize the concept of elevated central venous pressure (ECVP) load to all patients in critical care settings because of the limited cases and exclusive cohorts. Besides, the importance of ECVP duration was underestimated in estimating the ECVP load and analyzing its relationship with outcome, which is only accessible with the advantage of “big data” technology [12].

By using the large, public, de-identified clinical database, Multi-parameter Intelligent Monitoring in Intensive Care (MIMIC-III) [13], we evaluated the effect of ECVP on all patients in critical care settings, for whom CVP data were carefully recorded. We aimed to further characterize the association between mean CVP level and outcome as well as treatment received in the intensive care unit (ICU). Specifically, we investigated the potential effect of ECVP duration on outcome when mean CVP were stratified at different levels.

## Methods

### Data source

We conducted a large-scale, single-center, retrospective cohort study using data collected from the MIMIC-III open source clinical database (version 1.3, released on December 10, 2015), which was developed and maintained by the Massachusetts Institute of Technology (MIT), Philips Healthcare, and Beth Israel Deaconess Medical Center (BIDMC) [13]. Information derived from the electronic medical records of 46,476 unique critical care patients admitted to the ICUs at BIDMC between 2001 and 2012 was included in this free accessible database. Use of the MIMIC-III database has been approved by the Institutional Review Boards of BIDMC and MIT, and waiver of informed consent was granted.

### Patient population

All patients in the database were screened. The inclusion criteria in this study were as follows: (1) adults (≥18 years of age) at ICU admission, with complete medical records including available CVP measurement records; (2) ICU stay ≥72 h; and (3) consecutive CVP monitoring ≥12 h. For patients with multiple ICU stays, only data related the first ICU admission were considered.

All available CVP measurements recorded during ICU stay were extracted. Other variables including ICU type, demographic data, age, sex, Elixhauser comorbid conditions [14] and admission illness severity scores [including the Simplified Acute Physiology Score (SAPS) [15] and Sequential Organ Failure Assessment (SOFA) [16]] were extracted from the database. Additionally, data on use of vasopressors, mechanical ventilation, laboratory results related to organ dysfunction and length of ICU stay and hospitalization were extracted from the database.

### Exposure

The primary exposure was the mean CVP during the first 72 h after ICU admission. We also calculated the duration of ECVP_10_ as the time of CVP above the level of 10 mmHg, which was considered as a relatively higher CVP level that might by unbeneficial for patients in critical care settings [17], and used it as an alternative indicator of the mean CVP level to estimate the association between ECVP load and outcome in the critical care settings.

### Outcome

The primary outcome was 28-day mortality after ICU admission. The secondary outcomes included duration of mechanical ventilation and vasoactive drug use (epinephrine, norepinephrine, vasopressin, dopamine, dobutamine, milrinone and phenylephrine), laboratory results related to organ dysfunction and the length of ICU admission and hospitalization. For some patients, whose death occurred outside the hospital, the Social Security Death Index was linked to the database for investigations related to mortality.

### Statistical analysis

Baseline characteristics were stratified by quartiles of mean CVP during the first 72 h after ICU admission. Cox regression analyses were undertaken to compare the 28-day mortality among different mean CVP quartiles. All other covariates, which comprised demographic characteristics, ICU type, SAPS II score at admission, comorbidities and mean duration of CVP measurement, were included into the multivariable regression model. We also compared the survivors and non-survivors for their ECVP_10_ duration with the outcome independently through the stratification on mean CVP.

The results are expressed as mean ± SD (standard deviation) for normally distributed data or median [interquartile range (IQR)] for non-normally distributed data. Continuous variables were compared using one-way analysis of variance for normally distributed quantitative data, and Mann–Whitney *U* test for non-normally distributed quantitative data to determine differences between groups. All statistical analyses were performed by using the IBM^®^ SPSS^®^ Statistics version 22 (SPSS Inc., Chicago, IL, USA). Any *p* value <0.05 was considered statistically significant.

### Subset and sensitivity analyses

Since the mean CVP level can only describe the overall level of CVP, in sensitivity analyses, we replaced the mean CVP level with the duration of ECVP_10_ as an alternative indicator to estimate the association between ECVP load and outcome in the critical care settings.

We examined whether elevated mean CVP level in the highest quartile was associated with poor outcome compared to the other quartiles in patients with and without sepsis, which was defined as suspected or documented infection and an acute increase of ≥2 SOFA points infection [18], and AKI, as defined by either a greater than or equal to 0.3 mg/dL increase within 48 h or a greater than or equal to 50% increase within 7 days of ICU admission, or acute dialysis, in keeping with the Kidney Disease Outcome Quality Initiative guidelines [19]. We individually tested association between elevated mean CVP and these variables in adjusted analysis and provided graphical representation of the stratified risks.

## Results

### Baseline characteristics

**Fig. 1:**
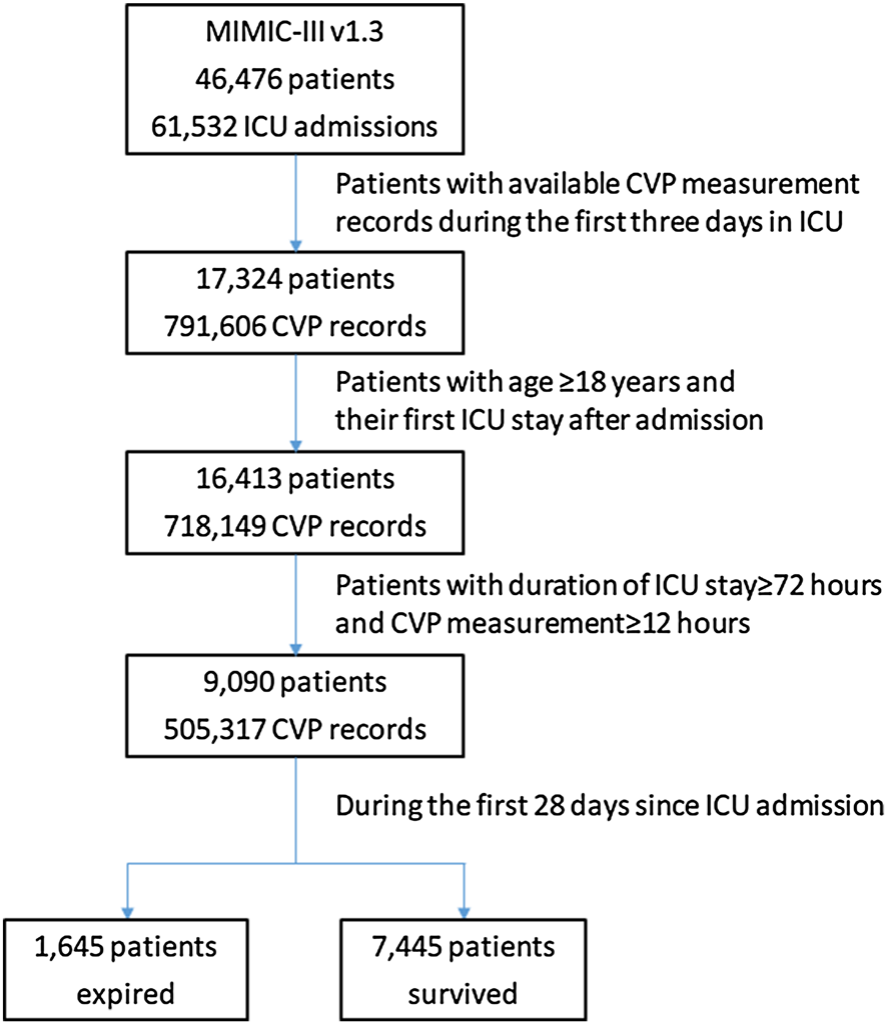
Flowchart showing step-by-step selection on patients included in the study. *CVP* central venous pressure, *ICU* intensive care unit

Among the 46,476 ICU patients and 61,532 ICU admissions in the MMIC-III v1.3, 17,324 patients underwent CVP measurement during the first 3 days of the ICU hospitalization and 791,606 CVP records were available in the database. Sequentially, we excluded 911 patients whose age at admission was below 18 years, 7323 patients with ICU stay less than 3 days and CVP measurement duration of less than 12 h, as illustrated in Fig. 1. The final cohort comprised 9090 patients with their first ICU admission and the corresponding 505,317 records of CVP measurement. The mean (±SD) interval between two consecutive CVP measurements was 0.9 ± 1.2 h, and the mean (±SD) duration of CVP measurement during the first 72 h in ICU hospitalization was 49.5 ± 18.8 h.

In the 9090 critically ill patients included in the study, the mean (± SD) CVP level was 11.8 ± 4.0 mmHg and median (IQR) ECVP_10_ duration of 23.0 (9.0, 43.8) h. As shown in Table 1, patients in the highest quartile of mean CVP level presented greater SOFA score and more comorbidities. The mean duration of CVP measurement and ECVP_10_ duration were also increased in higher quartiles of mean CVP level.

**Table 1:** General patient characteristics stratified by quartiles of mean CVP level during the first 72 h from ICU admission

	Quartiles of mean CVP level			
	1 (*n* = 2281)	2 (*n* = 2269)	3 (*n* = 2277)	4 (*n* = 2263)
Mean CVP/mmHg	7.4 (1.9)	10.6 (1.9)	13.1 (2.2)	17.4 (4.1)
Median CVP/mmHg	7	10	13	16
Age (years)	67.1 (15.8)	66.9 (15.9)	65.8 (15.5)	64.4 (15.4)
Sex, male	1070 (57.9%)	1088 (58.9%)	1046 (56.7%)	1007 (54.6%)
Ethnicity, *n* (%)				
Asian	33 (1.8%)	40 (2.2%)	21 (1.1%)	37 (2.0%)
Black	101 (5.5%)	114 (6.2%)	99 (5.4%)	119 (6.4%)
White	1326 (71.8%)	1301 (70.5%)	1297 (70.3%)	1293 (70.1%)
Latino	47 (2.5%)	41 (2.2%)	58 (3.1%)	57 (3.1%)
Other	341 (18.5%)	350 (19.0%)	370 (20.1%)	339 (18.4%)
ICU type				
Medical	437 (23.6%)	440 (23.8%)	481 (26.1%)	601 (32.6%)
Surgical	335 (18.1%)	238 (12.9%)	272 (14.7%)	293 (15.9%)
Trauma surgical	227 (12.3%)	198 (10.7%)	203 (11.0%)	167 (9.1%)
Cardiac surgery recovery	647 (35.0%)	739 (40.0%)	654 (35.4%)	558 (30.2%)
Coronary	202 (10.9%)	231 (12.5%)	235 (12.7%)	226 (12.2%)
Comorbidities				
Congestive heart failure	265 (14.3%)	277 (15.0%)	359 (19.5%)	363 (19.7%)
Cardiac arrhythmias	288 (15.6%)	291 (15.8%)	328 (17.8%)	408 (22.1%)
Hypertension	134 (7.3%)	170 (9.2%)	162 (8.8%)	659 (34.2%)
Valvular disease	88 (4.8%)	78 (4.2%)	117 (6.3%)	119 (6.4%)
Pulmonary circulation disease	63 (3.4%)	54 (2.9%)	78 (4.2%)	100 (5.4%)
Renal failure	167 (9.0%)	199 (10.8%)	202 (10.9%)	310 (16.8%)
Admission status				
Admission SAPS, points	20.6 (4.7)	21.2 (4.8)	21.9 (4.9)	23.0 (4.9)
Mean duration of CVP measurement (h)	40.55 (19.78)	22.88 (10.27)	51.25 (16.97)	60.24 (12.56)
Mean duration of ECVP_10_ (h)	5.65 (6.10)	20.60 (11.54)	35.70 (16.06)	47.90 (17.55)
ICU outcome				
28-day mortality (%)	15.4	16.8	17.3	22.8
Length of hospitalization (day)	15.4 (14.1)	15.4 (13.4)	16.1 (13.3)	17.8 (15.8)
Length of ICU stay (day)	6.9 (7.1)	7.8 (8.0)	9.3 (9.7)	11.0 (10.9)
Selected laboratory test				
WBC max, k/μL	19.1 (9.7)	19.9 (11.1)	20.4 (16.2)	21.4 (12.7)
Lactate max (mmol/L)	2.9 (2.6)	3.2 (3.1)	3.6 (3.2)	4.9 (4.5)
Creatinine max (mg/dL)	1.9 (1.9)	2.1 (2.0)	2.3 (3.5)	2.8 (2.7)
Total bilirubin max (μmol/L)	1.7 (4.8)	1.8 (4.3)	2.4 (5.9)	4.0 (8.2)
Treatment received				
Duration of vasopressor (day)	1.8 (4.4)	2.6 (5.4)	3.6 (7.1)	4.9 (8.4)
Duration of ventilator (day)	3.8 (6.9)	4.8 (8.0)	6.5 (9.7)	7.9 (11.0)
Fluid balance [L, Median (IQR)]	3.9 (0.7, 8.0)	4.6 (1.3, 9.0)	5.3 (2.0, 10.1)	6.8 (3.0, 12.8)

Values are presented as mean (SD), unless otherwise stated

*CVP* central venous pressure, *IQR* interquartile range, *ICU* intensive care unit, *WBC* white blood cell, *SAPS* Simplified Acute Physiology Score

### Mean CVP level and outcome

**Fig. 2:**
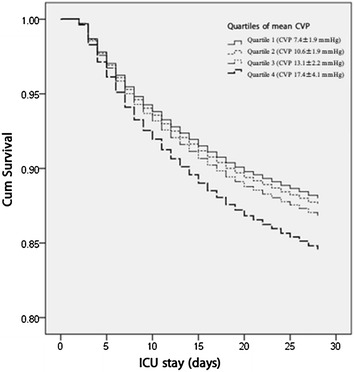
Survival curve of 28-day mortality by the quartiles of mean CVP level in the patients in the critical care settings. *CVP* central venous pressure

During the first 28 days after ICU admission, 1645 patients died. Higher quartiles of mean CVP level during the first 3 days after ICU admission were associated with higher unadjusted mortality. After adjusting for sex, age, gender, ethnicity, congestive heart failure, cardiac arrhythmias, hypertension, valvular disease, pulmonary circulation disease, renal failure and other 24 Elixhauser comorbidities, ICU type, SAPS II score at admission and duration of CVP measurement, the mean CVP level remained a significant predictor of 28-day mortality, as shown in Table 2. The other potential determinants are shown in Additional file 1: Supplement Table 1. The survival curve of 28-day mortality by the quartiles of mean CVP level is shown in Fig. 2, and the detailed comparison of 28-day mortality between the deciles of mean CVP level is shown in Additional file 1: Supplement Table 2 .

**Table 2:** Association between ECVP_10_ load during the first 3 days from ICU admission and mortality in 28 days

	Hazard ratio 28-Day mortality by quartiles of mean CVP level			
	Quartiles of mean CVP level			
	1	2	3	4
Mean CVP (SD)/mmHg	7.4 (1.9)	10.6 (1.9)	13.1 (2.2)	17.4 (4.1)
Deaths, *n* (%)	245 (13.3%)	273 (14.8%)	337 (17.5%)	433 (23.5%)
Unadjusted	1.00 (Ref.)	1.127 (0.949–1.340) *p* = 0.173	1.264 (1.068–1.496) *p* = 0.006	1.887 (1.613–2.207) *p* < 0.005
Adjusted*	1.00 (Ref.)	1.017 (0.848–1.219) *p* = 0.426	1.216 (1.018–1.451) *p* = 0.046	1.336 (1.117–1.599) *p* < 0.005

Hazard ratios (95% CI) provided. *Adjusted for sex, age, gender, ethnicity, congestive heart failure, cardiac arrhythmias, hypertension, valvular disease, pulmonary circulation disease, renal failure and other 24 Elixhauser comorbidities, ICU type, SAPS II score at admission and duration of CVP measurement

**Fig. 3:**
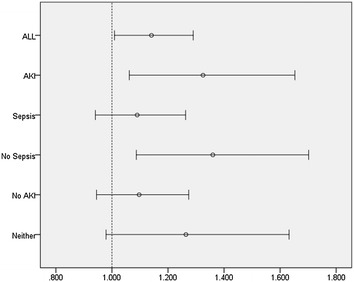
Forest plot for adjusted odds ratio value of higher mean CVP level (≥10 mmHg) and 28-day mortality per subgroup. Adjusted for age, gender, ethnicity, ICU type, congestive heart failure, cardiac arrhythmias, hypertension, valvular disease, pulmonary circulation disease, renal failure and other 24 Elixhauser comorbidities, ICU type, SAPS II score at admission and duration of CVP measurement. *AKI* acute kidney injury

In addition to the 28-day mortality, we investigated the association between quartiles of mean CVP level and secondary ICU outcomes, which included length of hospitalization, treatment received in ICU and laboratory results related to organ dysfunction, as shown in Table 1. The result showed that prolonged ICU stay and hospitalization were required for patients with higher mean CVP level during the first 3 days from ICU admission, and the duration of vasopressor or mechanical ventilation treatment also was prolonged. Furthermore, leukocytosis, higher serum bilirubin, creatinine, and lactate were all more commonly seen in the higher mean CVP quartiles.

### Subset and sensitivity analyses

Duration of ECVP_10_ was investigated between 28-day survival and non-survival patients. After stratification by different levels of mean CVP, as in Additional file 1: Supplement Figure 2, the results showed that the ECVP_10_ duration in non-survival group was significantly higher than in the survival group. Replacement of mean CVP with ECVP_10_ duration as indicator of ECVP load did not meaningfully change the association between ECVP load and 28-day mortality (odds ratio in the highest quartile, 1.354, 95% CI 1.151–1.594, *p* < 0.001).

To examine whether the higher mean CVP level remained associated with increased 28-day mortality across subset of patients, we explored multiplicative interaction terms with and without sepsis and AKI. No significant effect of ECVP on mortality was observed in patients with sepsis [*n* = 5121, odds ratio (OR) 1.090, 95% CI 0.941–1.263, *p* = 0.252] and without AKI [*n* = 6131, odds ratio (OR) 1.097, 95% CI 0.945–1.274, *p* = 0.224], while there was a significant trend toward increased mortality from higher ECVP load in patients without sepsis [*n* = 3969, odds ratio (OR) 1.36, 95% CI 1.087–1.702, *p* = 0.007], as illustrated in Fig. 3.

## Discussion

This study investigated the association between ECVP load and outcomes of patients in critical care setting. Based on comparison among patients with different levels of mean CVP, which is a measurement of ECVP load, we found that the higher mean CVP level was associated with increased 28-day mortality. Further investigation on length of hospitalization, duration of vasopressor treatment and mechanical ventilation, and laboratory results related to organ dysfunction also demonstrated that higher mean CVP level was associated with poor ICU outcome for patients in critical care settings.

In contrast to the common misleading statement of increasing CVP to a higher level to increase cardiac output, Starling curves, with the contribution of Guyton’s work, did not suggest any causal relationship between CVP and cardiac output, but emphasized the comprehensive role in the interaction between cardiac function and venous return of CVP. Recent studies have showed that not only the CVP failed as a useful measure for the assessment of preload and fluid responsiveness [20], but that the ECVP is independently associated with a higher mortality and increased risk of AKI in patients with sepsis and heart failure [7, 9, 11]. These results indicated that the CVP should be considered part of the evaluation in patients with hemodynamic instability. Furthermore, an ECVP may signify an impediment to venous return [3].

Based on the study for the consensus of congestive heart failure treatment, the pathophysiology of chronic systemic venous congestion should include decreased cardiac output from increased resistance to venous return, with the subsequent splanchnic damage and tissue perfusion insufficiency [1]. Integrated with the Starling–Guyton cardiac curve, the direct cause of ECVP may be defined as “the impediment to venous return by a relatively fatigued heart” [3]. Therefore, the potential causes of ECVP may include elevated venous return, cardiac dysfunction, increased vascular resistance, increased pericardial or intrapleural pressure, among other conditions. Our study showed that a higher ECVP_10_ load was associated with prolonged duration of treatment and length of ICU hospitalization. In sensitivity analysis, the result showed that impact of ECVP on mortality was limited in some subgroup patients, especially in the sepsis group, which was inconsistent with previous result [11]. Considering the difference in enrolling criteria, our result indicated that for the sepsis patients who survived the resuscitation phase, the benefit from successful resuscitation, which was always presented as higher CVP level, may attenuate the impact of ECVP on outcome and did not lead to statistically significant result. The fluid balance, mean CVP level and duration of ECVP10 result in different subgroups, and outcome (survival and non-survival) is shown in Additional file 1: Supplement Table 3.

In contrast to the long-term influence of systemic venous hypertension caused by cardiac dysfunction such as right heart failure, pericardial effusion and other conditions, the pathophysiological changes that occur with ECVP, as manifestation of systemic venous congestion, generally have acute consequences in patients being treated in critical care settings. Based upon the significance of association between ECVP load and ICU outcome, we also showed that the ECVP load should be considered and corrected in two independent aspects, level and duration, because both played important pathophysiological roles. According to the present results, we suggest that ECVP, with its association with worse outcome in critical care settings, should be considered seriously and further actions should be undertaken to discover potential causes and treatment. Our study was based on data extracted from electronic medical records in the MIMIC-III v1.3 [13], a large, open clinical database, which allowed precise research on ECVP load. To our knowledge, this study is the first to evaluate ECVP on a large population of patients managed in critical settings. More than 500,000 CVP records and 9000 patients were involved in our study. Furthermore, the use of database technologies and statistics played a critical role in reaching a meaningful conclusion in the present study. Given the extreme complexity and diversity of critically ill patients and their treatment, the traditional approach of knowledge discovery may not satisfy the need to address clinical concerns, and further efforts should be made to organize, share and analyze the “big data” in critical care [21].

This study has several limitations. First, our study is limited by its retrospective nature and the source of data used. For this reason, no causal relationships could be established. Additionally, CVP records could only be assessed in critical care patients with central venous catheter and we only extracted data from the first 3 days of ICU admission. Thus, our conclusions cannot be generalized to patients treated in a setting other than a critical care setting or without opportunities of continuous treatment during the first 3 days in ICU, for the latter of which extremely low or high CVP values may occur during the rescue/emergency phase of certain disease or pathophysiology process. Second, unlike the other studies on elevated mean CVP in certain patient populations and disease processes [5–9], we focused on a relatively long phase (72 h from ICU admission) during the general management of patients in critical care settings. Besides, it should be noted that we do not oppose to the significance of CVP targeting in some emergency events of critical care, such as the initial resuscitation phase of septic shock [2]. Third, although some predictors of illness severity were included in our study and adjusted analysis confirmed the association between ECVP_10_ load and poor outcome, our results may be affected by other confounding variables associated with organ damage or mortality. Additional prospective studies should be performed to investigate these parameters and the potential causes of ECVP load.

## Conclusion

In conclusion, this study showed that for patients in critical care settings, higher ECVP load was associated with poor outcome as well as prolonged treatment in ICU. Level and duration of ECVP should be evaluated, and more effort should be made to establish the cause and appropriate treatment of ECVP.

## Additional file

**Additional file 1.** Supplementary result for study on association between CVP and outcome

## Abbreviations

CVP: central venous pressure
ECVP: elevated CVP
ICU: intensive care unit
MIT: Massachusetts Institute of Technology
BIDMC: Beth Israel Deaconess Medical Center
MIMIC: Multi-parameter Intelligent Monitoring in Intensive Care
SAPS: Simplified Acute Physiology Score
SOFA: Sequential Organ Failure Assessment
IQR: interquartile range
SD: standard deviation
