# Supplementary material for: Association between elevated central venous pressure and outcomes in critically ill patients
Source: Ann Intensive Care. 2017 Aug 9;7:83. doi: 10.1186/s13613-017-0306-1 (PMC5549673; doi:10.1186/s13613-017-0306-1)
Supplement: Supplementary file 1 — Additional file 1. Supplementary result for study on association between CVP and outcome [file 13613_2017_306_MOESM1_ESM.docx]

eFigure 1. Histogram of the CVP value distribution in raw data (Panel A) and selected cohort (Panel B).

Abbreviation: CVP: central venous pressure.


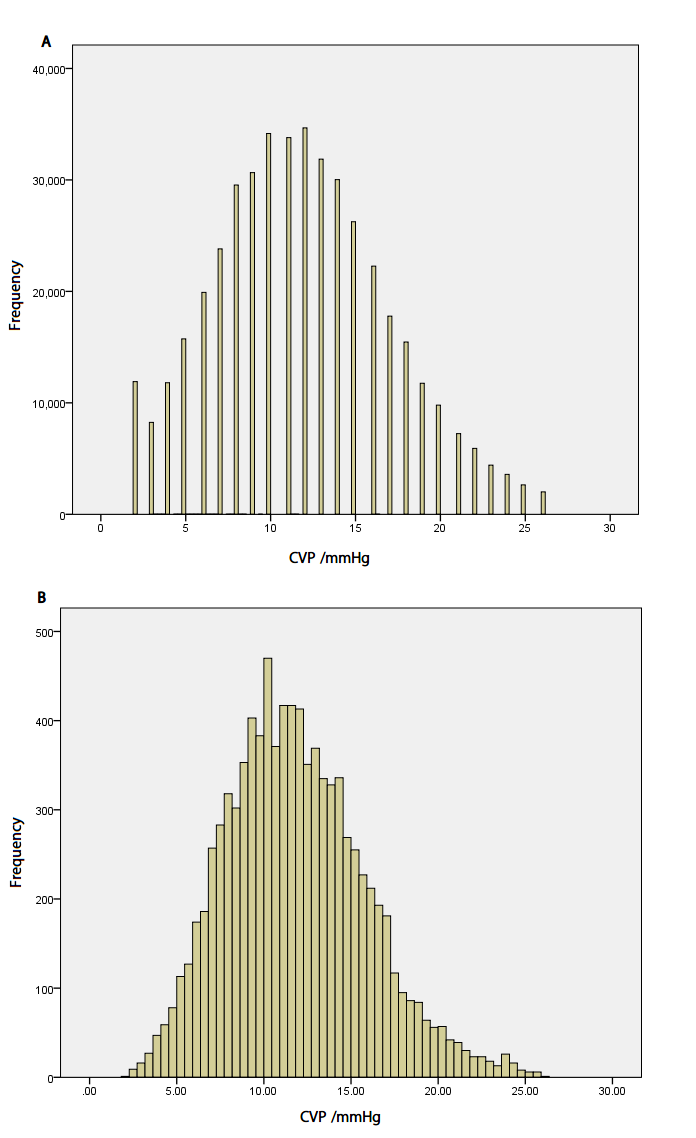


eFigure 2. Comparison of duration of ECVP10 between groups with different mean CVP(mmHg) and 28-day survival outcome. Panel A: comparison of ECVP10 duration between survival and non-survival group among different quartiles of mean CVP level, * p<0.005; Panel B: comparison of mean CVP level between survival and non-survival group among different quartiles of mean CVP.

Abbreviations: S, survival group; NS, non-survival group; CVP, central venous pressure; ECVP10, elevated central venous pressure above 10 mmHg;


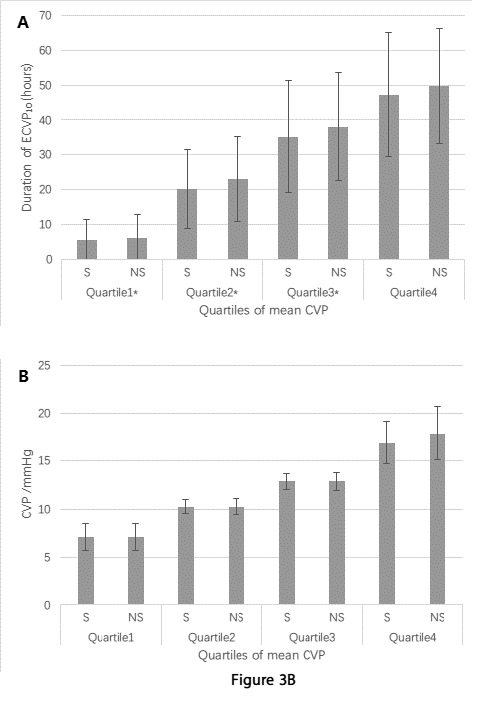


eFigure3 Survival curve of 28-day mortality by the quartiles of mean CVP level in the patients in the critical care settings in non-sepsis (A) and sepsis subgroup (B).

Abbreviation: CVP: central venous pressure.

**A**


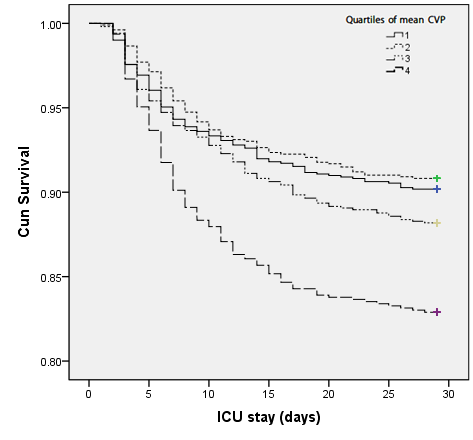


**B**


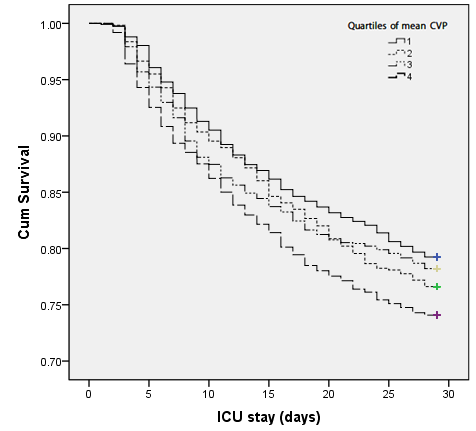


eTable 1. Multivariate Cox regression analysis of 28-day mortality in critically ill patients with different quartiles of mean CVP level during the first 72h from ICU admission.

| **Characteristic** | **Unadjusted Odds (95% CI), *p* Value** | **Adjusted Odds (95% CI), *p* Value** |
| --- | --- | --- |
| **Age** | 1.017 (1.014, 1.021), *p*<0.005 | 1.008 (1.004, 1.012), *p*<0.005 |
| **Admission SAPS II** | 1.044 (1.041, 1.047), *p*<0.005 | 1.032 (1.028, 1.036), *p*<0.005 |
| **Elevated mean CVP** | 1.259 (1.119, 1.417), *p*<0.005 | 1.141 (1.009, 1.290), *p*=0.035 |
| **Cardiac arrhythmia** | 1.944 (1.752, 2.157), *p*<0.005 | 1.200 (1.068, 1.349), *p*=0.002 |
| **Renal failure** | 1.476 (1.309, 1.664), *p*<0.005 | 1.396 (1.145, 1.701), *p*=0.001 |
| **Congestive heart failure** | 1.759 (1.584, 1.954), *p*<0.005 | 0.972 (0.862, 1.096), *p*=0.640 |
| **Valvular disease** | 1.582 (1.342, 1.864), *p*<0.005 | 1.066 (0.896, 1.267), *p*=0.472 |
| **Pulmonary circulation disease** | 1.582 (1.303, 1.922), *p*<0.005 | 1.148 (0.941, 1.401), *p*=0.174 |
| **Mean duration of CVP measurement** | 1.012 (1.009, 1.015), *p*<0.005 | 1.002 (0.999, 1.005), *p*=0.253 |

Abbreviation: CVP: central venous pressure; ICU, intensive care unit; SAPS, Simpliﬁed Acute Physiology Score.

eTable 2. 28-day Mortality between deciles of mean CVP level during the first 72h from ICU admission.

| Decile | 1 | 2 | 3 | 4 | 5 | 6 | 7 | 8 | 9 | 10 |
| --- | --- | --- | --- | --- | --- | --- | --- | --- | --- | --- |
| n | 912 | 912 | 910 | 902 | 914 | 912 | 903 | 911 | 906 | 908 |
| Death | 140 | 142 | 153 | 128 | 171 | 155 | 145 | 185 | 162 | 264 |
| Mortality % | 15.4 | 15.6 | 16.8 | 14.2 | 18.7 | 17 | 16.1 | 20.3 | 17.9 | 29.1 |
| Mean CVP (SD) | 5.6 (1.0) | 7.7 (0.4) | 9.0 (0.3) | 10.0 (0.3) | 11.0 (0.3) | 12.0 (0.3) | 13.2 (0.3) | 14.4 (0.4) | 16.0 (0.6) | 19.5 (2.1) |

eTable 3. Result of fluid balance, mean CVP level and duration of ECVP_10_ result in different subgroups and in outcome.

| **Group** | | **N** | **Fluid balance /mL** | **Mean CVP /mmHg** | **Duration of ECVP_10_ /hour** |
| --- | --- | --- | --- | --- | --- |
| **Sepsis** | **S** | 3,529 | 6,430 (2,647, 11,744) | 12.3 (4.0) | 29.8 (13.5, 50.5) |
|  | **NS** | 1,179 | 7,985 (3,992, 13,701) | 12.7 (4.4) | 31.0 (13.5, 51.7) |
| **AKI** | **S** | 2,319 | 7,175 (3,151, 13,224) | 12.6 (4.2) | 32.5 (15.3, 52.5) |
|  | **NS** | 640 | 8,811 (4,258, 14,936) | 13.2 (4.4) | 35.0 (16.7, 54.0) |
| **Non-sepsis** | **S** | 3,916 | 4,066 (425, 6,690) | 11.3 (3.6) | 18.3 (7.1, 36.8) |
|  | **NS** | 466 | 5,899 (2,435, 10,644) | 12.6 (4.6) | 29.3 (11.9, 50.5) |
| **Non-AKI** | **S** | 5,126 | 3,756 (688, 7,410) | 11.4 (3.6) | 19.5 (8.0, 39.0) |
|  | **NS** | 1,005 | 6,532 (3,036, 11,680) | 12.3 (4.4) | 27.4 (10.4, 48.9) |
| **All** | **S** | 7,445 | 4,606 (1,326, 9,203) | 11.8 (3.9) | 23.2 (9.3, 44.0) |
|  | **NS** | 1,645 | 7,409 (3,540, 12,841) | 12.7 (4.4) | 30.0 (13.0, 51.3) |

Abbreviation: S: Survival group; NS: Non-survival group; CVP: central venous pressure; AKI: acute kidney injury.
